# Characterization of starch and gum arabic-maltodextrin microparticles encapsulating acacia tannin extract and evaluation of their potential use in ruminant nutrition

**DOI:** 10.5713/ajas.18.0632

**Published:** 2018-11-28

**Authors:** Festus A. Adejoro, Abubeker Hassen, Mapitsi S. Thantsha

**Affiliations:** 1 Department of Animal and Wildlife Sciences, University of Pretoria, Pretoria 0028, South Africa; 2 Department of Biochemistry, Genetics and Microbiology, University of Pretoria, Pretoria 0028, South Africa

**Keywords:** Encapsulation, Gum Arabic, Maltodextrin, Starch, Rumen Fermentation, Tannin Extract

## Abstract

**Objective:**

The use of tannin extract and other phytochemicals as dietary additives in ruminants is becoming more popular due to their wide biological actions such as in methane mitigation, bypass of dietary protein, intestinal nematode control, among other uses. Unfortunately, some have strong astringency, low stability and bioavailability, and negatively affecting dry matter intake and digestibility. To circumvent these drawbacks, an effective delivery system may offer a promising approach to administer these extracts to the site where they are required. The objectives of this study were to encapsulate acacia tannin extract (ATE) with native starch and maltodextrin-gum arabic and to test the effect of encapsulation parameters on encapsulation efficiency, yield and morphology of the microparticles obtained as well as the effect on rumen *in vitro* gas production.

**Methods:**

The ATE was encapsulated with the wall materials, and the morphological features of freeze-dried microparticles were evaluated by scanning electron microscopy. The *in vitro* release pattern of microparticles in acetate buffer, simulating the rumen, and its effect on *in vitro* gas production was evaluated.

**Results:**

The morphological features revealed that maltodextrin/gum-arabic microparticles were irregular shaped, glossy and smaller, compared with those encapsulated with native starch, which were bigger, and more homogenous. Maltodextrin-gum arabic could be used up to 30% loading concentration compared with starch, which could not hold the core material beyond 15% loading capacity. Encapsulation efficiency ranged from 27.7%±6.4% to 48.8%±5.5% in starch and 56.1%±4.9% to 64.8%±2.8% in maltodextrin-gum arabic microparticles. Only a slight reduction in methane emission was recorded in encapsulated microparticles when compared with the samples containing only wall materials.

**Conclusion:**

Both encapsulated products exhibited the burst release pattern under the pH conditions and methane reduction associated with tannin was marginal. This is attributable to small loading percentages and therefore, other wall materials or encapsulation methods should be investigated.

## INTRODUCTION

The utilisation of tannins has become very important in ruminant nutrition studies as a result of their wide application in the ruminant animal production. Their biological significance has revealed that their protein binding properties can be harnessed in many applications to improve ruminant animal performance [1,2]. Dietary condensed tannin extracts have shown a significant reduction in enteric methane production both *in vitro* and *in vivo* [3]. This has been related to both direct inhibition of the growth of methane-producing archaea community (methanogens) through tanning action of their functional proteins, resulting in bacteriostatic and bactericidal effects or indirectly by the defaunating action on methanogen-associated protozoa populations [4]. Condensed tannins have also been found very effective in the control of intestinal parasites such as *Haemancorchotus conchortus* nematode and larva, both in ruminant species consuming tannins as part of their natural browse forage or by direct administration of the extract [3]. Other important applications of condensed tannins include the enrichment of conjugated linoleic acid in meat and milk via ruminal bio-hydrogenation, control of bloat and improving the efficiency of protein digestion via bypass of a good quality dietary protein [3].

Animal responses to dietary tannin have however been noted to be dependent on dose and other chemical characteristics of the tannin source [5]. Generally, increasing dietary condensed tannin concentration has resulted in a decrease in methane production per unit of digestible organic matter during rumen fermentation [2]. However, some of the limitations to the use of condensed tannins relate to their astringency and bitter taste, which among other negative consequences, leads to reduced voluntary dry matter (DM) intake in the animals [5]. The astringency of tannins occurs as a result of the interactions between polyphenols and salivary proteins, which results in precipitation of insoluble aggregates in the mouth thereby obstructing palate lubrication [6]. The administration of polyphenolic compounds like tannin extracts can be improved by the formulation of a finished product that is able to mask their taste while retaining their structural integrity until consumption, increase their bioavailability, and then deliver as well as release them precisely at the target site [7]. This can be attained through the various encapsulation techniques [7].

The microencapsulation process relies on the use of wall materials that are biological polymers [7]. Various wall materials can be utilised in encapsulating plant extracts and various polyphenolic substances in the food/feed industry. These wall materials include among others, starch, maltodextrin, gelatine etc. or combination of polymers such as maltodextrin and inulin, maltodextrin and gum arabic, gum arabic and tapioca starch [8] depending on the characteristic properties of the active ingredient. Additives for ruminant animals that have been widely encapsulated include rumen-protected amino acids (methionine, lysine), multivitamin products, fumaric acid and slow release urea products [9]. However, the limitations of cost and suitability of many of the common polymers used in the food industry have been noted and may hinder their commercial use in livestock applications. Their pattern of release of the active ingredient in the ruminant digestive system has also not been extensively evaluated. Therefore, the selection of a suitable wall material is critical to the success of the encapsulation process in terms of efficiency, yield and retention of the biological activity of the core material [10].

The effectiveness of gum arabic in the encapsulation of polyphenolic extracts has been documented in literature but remains an expensive choice in most food applications [11]. Starch is a common wall material, though with very low emulsifying properties but very cheap and accessible [11]. Maltodextrin, a hydrolysed starch product also offers the advantage of being cheap, has low viscosity at high solid concentrations and capable of protecting the core material against oxidative damage [12]. Encapsulation of tannin extract has the potential of reducing the impact of tannin consumption on DM intake. Besides, a sustained release of tannin in the rumen will also improve its utilisation significantly. This study aimed to encapsulate acacia tannin extract (ATE) with native starch or maltodextrin-gum Arabic and then characterize the microparticles based on their morphology, encapsulation efficiency (EE) and yield. Furthermore, the *in vitro* release profiles of the microparticles in buffer solutions that simulate ruminant gastrointestinal tract, as well as effect of their dietary supplementation on *in vitro* gas production were studied. To the best of our knowledge, there are no published works reporting the encapsulation of tannin extract for ruminant animal applications, specifically using these wall materials.

## MATERIALS AND METHODS

### Animal care

This study was carried out in accordance with the guidelines stipulated by the National Health Research Ethics Council of South Africa and approved by the University of Pretoria Animal Ethics Committee (AEC) with the approval number EC061-14.

### Materials

The ATE, a water-soluble extract from the *Acacia mearnsii* tree bark was obtained from UCL Tannin Pty (Ltd), South Africa, and used as the active ingredient or core material in the current study. Gum arabic, maltodextrin (DE 16.5), native potato starch and tannic acid were obtained from Sigma Aldrich Inc. (St. Louis, MO, USA); the F57 fibre filter bags were purchased from ANKOM Inc. (Fairport, NY, USA). All chemicals and reagents were of analytical grade in purity.

### Properties of acacia tannin extract

The ATE, obtained from UCL Tannin Company Pty (Ltd), Dalton, South Africa, was extracted from the bark of black wattle (*Acacia mearnsii*) tree by steam distillation, and then concentrated into powdered form. The extract has a molecular weight that ranges from 500 to 3,000, with an average of 1,250 and it contains other non-tannins (including low molecular weight polyphenols, salts, sugars, and organic acids). The result of our laboratory analysis showed that the sample had total phenol and total tannin concentrations of 65.8% and 58.5% respectively (as tannic acid equivalent) according to the procedure of Makkar et al [13] and condensed tannin concentration of 30.5% (as leucocyanidin equivalent) according to the procedure of Porter et al [14]. Because of possible variation in extracts’ characteristics from the company, a single batch of extract, stored at 4°C was used for all preparations and analysis throughout this study.

### Microparticles preparation

The microparticles were prepared using a procedure similar to that of Zhang et al [8] with slight modifications. Briefly, wall materials were dissolved in water and homogenised. The ATE was added to the solution under continuous stirring and homogenization using an overhead homogenizer for 180 seconds (RW 20, Ika-Werke, Janke & Kunkel-Str. 10, Staufen, Germany). For the maltodextrin-Gum Arabic microparticles, the following parameters were fixed for all preparations: Gum-Arabic and Maltodextrin were added at the ratio of 40:60 (w/w); solute concentration in water was 1% (w/v) [9]. For the starch microparticles, native starch was suspended in ethanol:water (10:90 v/v), and heated under continuous mixing at 65°C to 70°C until a gelatinised paste was obtained. Gradual cooling was done with the addition of tannin extract under continuous homogenization as described by Fernandes et al [10]. The final mix was freeze-dried, and thereafter stored away in an airtight container. The ratio of wall to the core material in both preparations was varied from 85:15 to 65:35 in order to evaluate optimum loading conditions. The different preparation concentration of the wall material and ATE are designated as maltodextrin-gum Arabic tannin extract 25–35 (MG-TE_25–35_) and starch-tannin extract 15–30 (S-TE_15–30_).

### Spectrophotometric analysis of tannin extract

The total phenol content of the ATE was evaluated using the Folin-Ciocateau colorimetric method and values were expressed as tannic acid equivalent [14] while the total condensed tannin content was evaluated using the butanol-HCl method and expressed as leucocyanidin equivalent [14]. For assaying the total phenol content, about 0.5 mL of extract was vortex mixed with 0.25 mL of the Folin-Ciocateau reagent and 1.25 mL of 20% sodium carbonate. The absorbance of the resultant solution was measured at 725 nm after 40 min and concentration estimated using a standard absorbance curve from tannic acid. Total polyphenolic contents were expressed as mg/g tannic acid equivalent. For the butanol-HCl method, 0.5 mL of sample containing extract, 3.0 mL butanol HCL (95:5 v/v) and 0.1 mL ferric reagent was added. Samples were vortexed and heated at 100°C for 60 min and absorbance read at 550 nm. A suitable blank containing the unheated sample was subtracted. For the quantification of EE, and rate of release of the active ingredient from microcapsules, the Folin-Ciocateau method was used as it allows for easy reference with a known standard, in this case, tannic acid [13]. Blank microcapsules were used to correct for the effect of wall material on the absorbance readings. Freeze-dried microparticles were evaluated by quantifying the amount of the bioactive compound on the surface of microparticles and the total amount of the bioactive compound loaded as described previously [8,14].

#### Surface tannin content

Surface tannin (ST) was estimated as the amount of un-trapped ATE in the microparticles. Briefly, starch microparticles (200 mg) were dispersed in 20 mL of an ethanol:methanol solution (1:1) and filtered through a 0.22 μm Millipore filter membrane while for gum arabic/maltodextrin matrix, 200 mg of microparticles was severally washed with a total of 45 mL anhydrous ethanol, filtered and the filtrate made to 50 mL mark with distilled water. An aliquot was taken to determine the ST content of the encapsulated product.

#### Total tannin content

The structure of the coating material for each microparticle was completely destroyed for evaluation of total bioactive compound loaded as against the theoretical amount added. For starch microparticles, 200 mg of the samples were dispersed in 20 mL of 52% (aqueous) perchloric acid and ultra-sonicated for 15 min and aliquots were taken for analysis [11]. For the maltodextrin/gum-arabic matrix, 200 mg sample was macerated with pestle and mortar with 5 mL distilled water and thereafter washed with anhydrous ethanol and filtered. Filtrate collected was made to 50 mL volume and aliquot sampled for analysis of tannin content.

#### Determination of core loading and encapsulation efficiency

ST percentage (ST %) and the EE were calculated according to equations 1 and 2, respectively [11].

(1) $$ST\;(\%)=\left(\frac{surface\;tannin\;concentration}{total\;tannin\;recovered}\right)\times 100$$

(2) $$\text{EE }(\%)=100\times (\frac{total\;tannin\;recovered-surface\;tannin\;concentration}{Theoretical\;loaded\;tannin\;concentration})$$

#### Morphological analysis of microparticles using scanning electron microscopy

Morphology of microparticles and particles sizes were evaluated by viewing with a scanning electron microscope. Preparations of microparticles were mounted on a slide with double-sided tape and coated with carbon before sputtering with gold under an argon atmosphere using an Emitech K950X (Ashford, UK) vacuum carbon evaporator. The gold sputtered microparticles were then viewed under a field emission scanning electron microscope (FE-SEM), ZEISS ULTRA PLUS (JEOL, Tokyo, Japan). The size of the microparticles was determined by comparing the images of at least 50 microparticles per treatment, with those of a scale bar of the same magnification.

### *In vitro* release kinetics of encapsulated Acacia tannin from microparticles

The *in vitro* release of tannin extract from the encapsulation matrix in the digestive system of ruminant animals was simulated using product solubility in various pH media following the procedure of Rossi et al [15]. Elution media used were: acetate buffer (pH 5.6), HCl buffer (pH 2.2) and phosphate buffer (pH 7.4) to simulate rumen, abomasal and intestinal conditions, respectively. In this study, the F57 ANKOM filter bags with a porosity of 25 μm used in fibre analysis was used. Microparticles (200 mg) were placed in the fibre filter bags and sealed using bag sealer, thereafter suspended in 50 mL of elution media, and agitated at 50 rpm inside an incubator at 39°C [11]. Aliquots of 1 mL were removed at 30 min, and at 1, 2, 4, 8, and 24 hours after incubation for analysis. The initial volume was maintained by the addition of fresh media. Aliquots taken were frozen immediately for subsequent analysis. The release of tannin was monitored by UV spectrophotometry as described above. The cumulative amount of tannin released at each time interval was corrected with the volume of the dissolution media. The data obtained for acetate was further fitted into zero order, first order, and Higuchi *in vitro* release kinetic models to find the best which predict the release of tannin extract in the rumen [16].

### *In vitro* gas production

*In vitro* gas production study was performed following the procedure of Menke et al [17] with the modifications detailed in Adejoro and Hassen [18]. Rumen liquor was collected from two rumen-cannulated merino wethers fed lucerne hay (*Medicago sativa*) *ad libitum*, strained through four layers of cheesecloth into a pre-warmed thermos flask, and transported quickly to the laboratory. This rumen fluid was mixed with buffer-mineral solution and added to each serum bottle, which already contained approximately 400 mg of substrate, under a continuous stream of CO_2_ and sealed with rubber stoppers. All bottles were placed inside an incubator at 39°C and 120 rpm. Using a semi-automated gas pressure transducer and digital tracker, gas pressure was recorded at 2, 4, 8, 12, and 24 h after incubation, and which was subsequently converted to volume. Gas samples were collected using a syringe and analyzed for methane concentration using gas chromatography (8610C BTU Gas analyser GC System, SRI Instruments, Bad Honnef, Germany). *Eragrostis curvula* hay (crude protein [CP], 55 g/kg; neutral detergent fibre [NDF], 784 g/kg; acid detergent fibre [ADF], 492 g/kg) and a total mixed ration (TMR) diet (CP, 180 g/kg; NDF, 301 g/kg; ADF, 214 g/kg) were used as substrates, and incubated with the crude ATE, or encapsulated ATE. The incubations containing an equivalent amount of the wall materials only (starch or maltodextrin/gum-arabic) were also included for comparison. For each substrate, treatments included i) diet only (control), ii) diet plus crude ATE, iii) diet plus starch-encapsulated ATE, iv) diet plus gum arabic-maltodextrin-encapsulated ATE, v) diet plus starch only, vi) diet plus gum Arabic-maltodextrin only. To each treatment containing ATE or encapsulated-ATE was added an equivalent of 25 mg ATE, which corresponds to 2.33% CT (leucocyanidin equivalent). Gas production data was fitted into the Ørskov and McDonald equation *y* = b(1–*e*^−^*^ct^*) [19] to predict the rate and extent of fermentation (y = gas production at time t; b = slowly fermentable fraction, mL/g DM; and c = rate of fermentation of fraction ‘b’, mL/h). Rumen fluid pH was measured after 24 hours of incubation using a pH meter (Mettler Toledo 230 pH meter, Mettler-Toledo, Powai Mumbai, India) while ammonia-nitrogen concentration was analysed spectrophotometrically.

### Statistical analysis

Results of microparticles sizes yield and EE were expressed as mean of at least four repeat batches. For the *in vitro* gas production, individual bottles within each run served as analytical replicates and were averaged prior to statistical analysis, while each run served as a statistical replicate. Gas volume was plotted against incubation time using the non-linear equation to predict fermentation kinetics [19]. Data on morphological parameters and gas production were analysed using the general linear model (GLM) procedure of SAS 9.3 (SAS Institute Inc., Cary, NC, USA) with the model:

$${\text{Y}}_{\text{ij}}=\mu +{\text{B}}_{\text{i}}+{\text{T}}_{\text{j}}+{\text{e}}_{\text{ij}}$$

Where, μ = overall mean, B_i_ = block effect (replicate), T_j_ = treatment effect, Y_ij_ = mean of individual observation and e_ij_ = residual error. Mean separation was done using Tukey’s test and significance was declared at p<0.05.

## RESULTS

### Characterization of encapsulated acacia tannin microparticles

**Figure 1:**
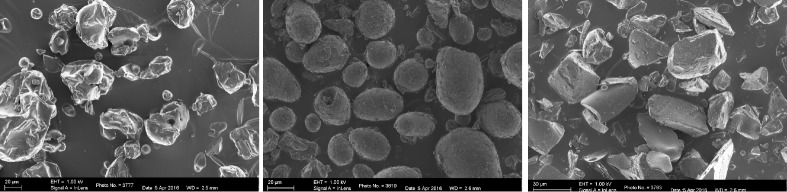
Electron micrographs showing the external morphological characteristics of un-encapsulated acacia tannin extract (A), encapsulated with native starch (B), and maltodextrin-gum-arabic (C) as viewed under field emission scanning electron microscope. At a scale bar at 20 μm, unencapsulated tannin extract revealed irregular shaped particles while starch encapsulated microparticles were bigger and most spherical in shape. On the other hand, maltodextrin-gum Arabic encapsulated microparticles showed flake-like structures.

Scanning electron microscopy showed that the unencapsulated ATE particles were irregular in shape and had shiny or glossy surfaces with numerous impregnations (Figure 1). Their particle size ranged from 15 to 40 μm diameter, with most particles within the 20 to 25 μm range. Native starch (S-TE) and maltodextrin-gum arabic (MG-TE) encapsulated ATE ranged in size from 20 to 65 μm in diameter, with S-TE having more particles within 30 to 45 μm while MG-TE particles were mostly within 25 to 40 μm. The native S-TE microparticles were slightly bigger than the MG-TE microparticles. The S-TE microparticles were generally spherical or ovoid shaped, and dull in appearance while the MG-TE microparticles appeared in various flake shapes with mostly flat surfaces. There was a discernible effect of encapsulation on microparticle morphology, with the majority of them having a diameter of 25 to 40 μm, and the outer surfaces showing obvious modifications and evidence of adherence of the wall materials to the ATE particles in the form of several layers of coating.

### Yield and encapsulation efficiency of microparticles

In this study, the core (ATE) to wall material ratio was evaluated as the independent variable, capable of affecting the yield and EE of ATE. There were significant differences in surface and total tannin contents among the wall materials used, within each wall material (Table 1). The ratio of core to wall material also significantly influenced the surface and total tannin contents of microparticles. The ST contents of the microparticles ranged from 35.0% to 63.9% for S-TE and 8.43% to 11.2% for MG-TE. The total tannin recovered varied from 70.9% to 75.8% and 63.2% to 70.7% for S-TE and MG-TE microparticles, respectively (Table 1). The ST contents in MG-TE microparticles was generally lower than that in S-TE. Within each wall material, when the concentration of ATE was varied, total tannin recovered was not significantly different in both MG-TE and S-TE microparticles. The result of EE for MG-TE showed values of 64.8% at 25:75 (core to wall material ratio), 60.5% at 30:70 and 56.1% at 35:65. There was a decrease in EE with increasing concentration of ATE. For S-TE, the result showed that at 15% ATE concentration, EE was 48.8% while 33.4%, 27.7%, and 31.1% were recorded at 20%, 25%, and 30% ATE concentrations, respectively (Table 1).

### *In vitro* release of acacia tannin from microparticles in dissolution media

**Figure 2:**
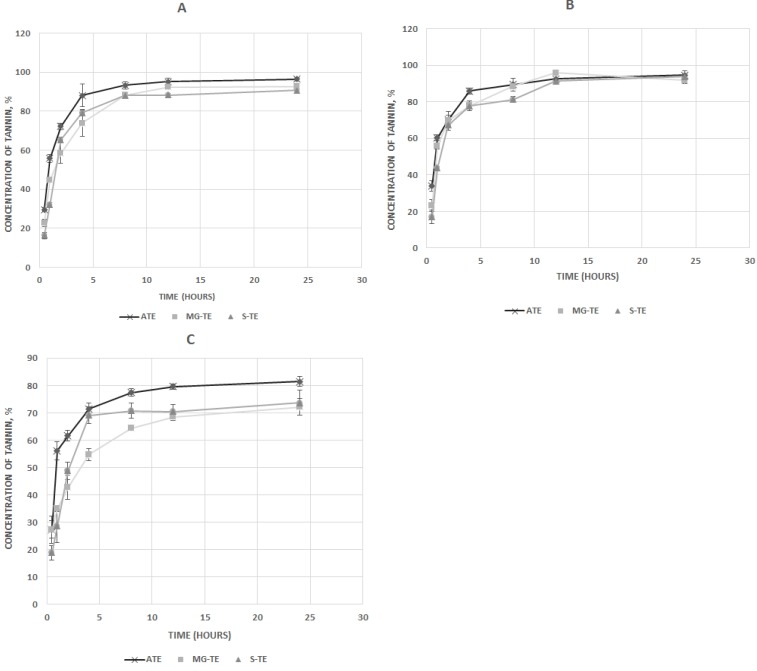
*In vitro* release profile of acacia tannin extract microparticles in (A) acetate buffer, pH 5.6; (B) phosphate buffer, pH 7.4; (C) HCl buffer, pH 2.2, at 39°C. ATE, unencapsulated tannin extract; S-TE, ATE encapsulated in starch (S-TE); MG-TE, ATE encapsulated in maltodextrin/gun-arabic.

The release profile of tannin from the unencapsulated ATE, S-TE, and MG-TE under optimal loading conditions, in acetate, phosphate and HCl buffer media at 39°C are shown in Figure 2a–c. In each dissolution media, a burst release pattern was observed within the first 4 hours of incubation for all microparticles across the different pH media. This phase was followed by a phase of gradual release from 4 to 8 hours after dissolution. Beyond, 8 hours, most of the tannin in the microparticles had been released into the dissolution media. For the unencapsulated ATE, about 75.5% of ATE was released within 2 hours and about 90.2% within 4 h after dissolution at 39°C. The release profile indicates that 58.7% and 75.8% of tannin was released from the MG-TE microparticles at 2 hours and 4 hours, respectively while 65% and 78.7% were released from S-TE after the same incubation times respectively. The *in vitro* release data obtained in acetate buffer, simulating rumen pH, showed that the release patterns of tannin from microparticles fitted into the Higuchi and first-order models better than the zero-order model (Table 2).

### Effect of acacia tannin extract encapsulated with starch or maltodextrin-gum arabic on *in vitro* ruminal gas production and methane emission

The ATE treatment reduced total gas production up to 24 h incubation when compared to the control diet in both substrates (p<0.0001). The inclusion of S-TE and MG-TE generally resulted in higher total gas production across the time intervals up to 24 h when compared with the control treatment in both *Eragrostis curvula* (EC) and TMR substrates. However, total gas production was lower in S-TE and MG-TE treatments than in incubations containing only the wall materials (Starch; gum arabic/maltodextrin) (p<0.0001). The ATE tended to reduce methane emission in both EC and TMR substrates (p<0.0001). The S-TE and MG-TE incubations did not reduce methane production when compared with the control or ATE treatment in both substrates (p<0.0001), however, in the TMR incubations, methane values obtained in S-TE were lower compared to the starch only treatment (p<0.0001). The intensity of methane produced when expressed as the ratio of methane volume to total gas volume showed that tannin inclusions did not affect methane concentration across the treatments in EC (p = 0.38) and TMR (p = 0.13) substrates. The rate of fermentation of the insoluble fraction with starch and S-TE was higher compared to the control, ATE and incubations with gum Arabic and maltodextrin in the Eragrostis hay substrate (p<0.0001). However, no difference in fermentation kinetics across the treatments was observed in the TMR substrate (p = 0.68). Supplementation with ATE, S-TE, or MG-TE did not affect rumen pH after 24 h incubation in both Eragrostis hay (p = 0.15) and TMR (p = 0.11) substrates. However, with Eragrostis hay as the substrate, ATE, S-TE, and MG-TE reduced ammonia nitrogen concentration in rumen fluid after 24 h *in vitro* incubation (p = 0.04) while no differences were observed in the TMR substrate (p = 0.11). The unencapsulated ATE was more effective in reducing total gas and CH_4_ production than samples containing S-TE and MG-TE (Table 3).

## DISCUSSION

The variations in microparticles size as observed in this study are likely due to the influence of the size of core materials, the encapsulation method used and the molecular sizes of the wall material [20], which will ultimately influence the EE of the microparticles. The various chip/crumb-like shapes formed by MG-TE microparticles may be associated with the method of dehydration (freeze-drying) or the properties of the wall materials [21]. The surface morphological characteristics of the MG-TE microparticles observed were similar to the description of microparticles reported earlier as flake-like structures, free of dents and shrinkage when gum-arabic/sucrose/gelatine was used as wall material in encapsulating limonene under freeze-drying conditions [22] or when lemon pomace aqueous extract was encapsulated with maltodextrin under freeze-drying conditions [12]. A broken-glass shaped structure was similarly observed when gum-arabic was used to encapsulate garcinia fruit extract using freeze-drying method [21]. Agglomeration and stickiness of particles often noted as a limitation when using maltodextrin as a wall material in the previous study [21], was however not observed in the current study. This may be an indication that the combination of gum-arabic with maltodextrin served as a more effective wall material for encapsulation of ATE. Smooth microparticles that are round shaped and devoid of dents, in native starch encapsulated beta-carotene powders were observed by Loksuwan [23] and this is in agreement with the scanning electron micrograph observations of S-TE microparticles in this study. The encapsulation process may have provided enough opportunity for the starch to interact with the tannin molecules. Pre-gelatinisation of starch has been found to improve its ability to encapsulate polyphenolic core materials [23]. Pitchaon et al [24] observed that a combination of maltodextrin and gum Arabic produced better encapsulation for phenolic antioxidants, with higher EE.

The results of this study showed that beyond 30:70 ratio of core to wall material, EE decreased significantly for the MG-TE microparticles while for S-TE microparticles, the significant decline occurred beyond 15:85 ratio. The interaction of the active ingredient (core material) and wall material seems to have a profound effect on encapsulation parameters, specifically the loading capacity and EE. The proportion and nature of the core material in the total microparticles have been noted as very important factors influencing the efficiency of microencapsulation and the overall application of an encapsulated product. Previous research by Fernandes et al [10] with starch, maltodextrin, maltodextrin-gum Arabic and gum arabic reported EE values ranging from 45.45% to 60.22% in encapsulating a lipophilic core material while Robert et al [11] reported values ranging from 47% to 61% when starch or acetylated starch was used for encapsulation of gallic acid, a hydrophilic polyphenolic compound. However, very high EE of 99.2% and an encapsulation yield of 89.71% were previously reported for maltodextrin-gum Arabic microparticles encapsulating grape seed extract [8].

Core material concentration, as a factor, affected the EE of the microparticles in this study just as it had been earlier reported [8]. The higher loading capacity and EE obtained in the maltodextrin-gum arabic microparticles compared to native starch may be related to the structural differences of the wall materials, leading to a probable higher binding capacity of maltodextrin-gum arabic combination over native starch. The result of this study showed that this wall material combination was effective for the tannin extract but not beyond 30% of the core material. Similarly, high EE for maltodextrin-gum Arabic microparticles encapsulating phenolic antioxidants has been reported [24]. Encapsulation efficiencies in maltodextrin and gum Arabic as encapsulating agents were higher than native starch with Acai powder core material, although the spray-drying method was applied in that study [20]. The properties of wall materials have been noted as an important factor that affects EE [10]. The better entrapment of ATE in the maltodextrin-gum arabic microparticles can be linked to the plasticity of gum arabic which is capable of forming a good film over the core material, and thus prevents the cracking of the matrix [25]. Encapsulation method also has significant effects on the extent and efficiency of encapsulation. When gum arabic was used as a wall material in preparation of spray dried microparticles, shrinking and denting of microparticles because of the evaporative dehydration process was observed. Spray-drying as an encapsulation method has been reported to result in the formation of spherical microparticles with concavities when maltodextrin was included as wall material. This was attributed to the shrinkage of particles due to rapid moisture loss after cooling. In contrast, in that study, microparticles prepared by freeze-drying produced flake-like shape devoid of indentations and could be associated with the lack of forces to break up the frozen liquid into droplets [12].

The initial rapid release of tannin in both S-TE and MG-TE may be attributed to the presence of surface (uncoated) tannin as well as the encapsulation properties which can be affected by wall material properties, the interaction between the wall and core materials, and method of encapsulation [11]. This is an indication that these microparticles may be easily solubilised in the mouth or rumen of the ruminant animals. The solubility of starch in aqueous media and the tightly bound ATE particles to wall materials and its gradual erosion may have influenced the second phase of ATE release. In the HCl buffer, a lower release of ATE was observed even after 8 h of dissolution of microparticles. Tannin dissociation from existing bonds has been known to depend on pH [26]. During the gelatinisation process, some amylose content of starch, which carries a functional group capable of attaching to tannins, may be leached into solution [27]. The interaction of amylose with tannins have been observed to slow starch retrogradation after gelatinization and reduce its rate of *in vitro* degradation due to the formation of stronger hydrogen bonds [27]. However, various modifications of starch such as high amylose starch, and other modified starch products like acetylated starch, have been found to further improve its binding ability. Similarly, it has been found that the protein binding activity of tannins can be affected by the presence of other polysaccharides such as pectin, gum arabic, carrageenan, xanthan, and gellan [28]. The ability of these polysaccharides to form hydrophobic pockets and encapsulate polyphenols have been observed to result from the formation of hydrogen bonds between the oxygen atom of the carbohydrates and the hydroxyl group of the tannin [6].

The effect of ATE on *in vitro* ruminal gas production obtained in this study is consistent with previous studies involving the use of ATE in reducing total gas and CH_4_ production [2]. However, encapsulating ATE with starch or gum arabic and maltodextrin rather than reducing methane, it triggered an increase in methane and gas production *in vitro*. This can be attributed to the high concentration of the encapsulating materials (starch, maltodextrin, gum arabic) which are potentially fermentable and thus might have acted as substrates for rumen microbes. Significantly higher methane production in sheep can be associated with increased NDF digested [29]. When rumen ammonia nitrogen concentration is adequate, increase in fermentable carbohydrate results in greater microbial growth, and consequent increase in fermentation and gas production [17,30]. Substrate type, the chemical nature of tannin, and its concentration in diet, as well as the concentration of tannin, may influence its protein binding biological activity, antimethanogenic effect and rumen microbial function and subsequently, methane production, or nutrient digestibility [29].

Tannins are known to reduce total gas and methane production by a reduction in methanogenic activities, the overall reduction in fermentation or a combination of both [5]. The impact of the tannin inclusions in this study showed that tannin extracts in ATE, S-TE, and MG-TE might have reduced organic matter fermentation rather than exert any specific effect on methanogenesis. This result is similar to previous studies [31] where condensed tannin extracts exert significant reduction on DM disappearance and gas production, as a consequence of reduced fibre degradation. Reduced fibre digestion may have negative consequences, especially when animals are consuming poor quality roughages such as EC and a compromise on digestibility may affect nutrient intake and performance [31]. The supplementation of ATE, S-TE, and MG-TE did not affect the rate of fermentation in the TMR substrate although the addition of starch, as an encapsulating material, significantly increased fermentation rate in the EC substrate. Generally, the impact of tannins or tanniferous plants on substrate fermentation rate varies for various tannin sources [32]. While some researchers have reported a significant reduction in the rate of substrate fermentation [3], others have observed no such effect [33]. This may largely be due to the varying properties of the tannins or other diet characteristics. Tannin sources which reduce methane production but exhibited only minor impact on gas production have better potential at being exploited as antimethanogenic supplements [32].

Where the nutrient requirement of animals is adequately supplied, reduced ruminal CP degradation offers the potential bypass of dietary protein to the lower part of the digestive system. A shift in protein digestion to the hindgut may be advantageous to the animal and also, the reduced urinary N loss as opposed to faecal N loss has potential environmental benefits [1]. The presence of tannin extract is often associated with reduced protein degradability in the rumen, often resulting in a lower concentration of ammonia nitrogen [31]. Although reduced nitrogen degradation resulting in lower ammonia nitrogen is common with tannin supplementation [30], in the TMR diet, ammonia nitrogen concentration between S-TE, MG-TE, and ATE was not different and this is an indication that encapsulation may not have affected the impact of tannin on protein degradation in the rumen after 24 h. In the EC, a substrate with lower CP content, S-TE resulted in reduced ammonia nitrogen concentration, a pattern that has been widely reported in previous reports [26,29].

The high concentration of potentially degradable materials in the encapsulation wall materials may have exerted a confounding effect on the impact of tannin extract or the encapsulated tannin on rumen proteolysis and other fermentation characteristics. Therefore, the level of inclusion of these materials in encapsulating ATE posed limitations to its application in methane mitigation studies. This could be partly due to the low loading percentage of the tannin within the wall materials, the encapsulation process or the potential of the wall materials to serve as a source of fermentable energy for rumen microbes. Further studies are therefore needed, to evaluate the effect of S-TE and MG-TE in other ruminant applications while other encapsulation techniques may be explored for tannin utilisation when gas production and methane emission are of interest.

## CONCLUSION

Starch and maltodextrin-gum Arabic were successfully used in encapsulating ATE. The maltodextrin/gum-arabic microparticles were smaller and more homogenous than those of native starch even at higher loading concentration. Microparticles produced using both wall materials exhibited the burst release profile under various pH conditions. In terms of methane production, encapsulated microparticles showed an only slight reduction in methane when compared with the samples containing only the wall materials but methane production was generally higher than in the unencapsulated tannin extract. These encapsulated microparticles need to be tested in other ruminant applications while other encapsulation methods suitable for tannins in enteric methane mitigation need to be developed.

## Figures and Tables

**Table 1:** Effect of variation in processing parameters on encapsulation efficiency and recovery of acacia tannin extract in starch and maltodextrin-gum arabic microparticles

Sample1)	Wall material (g/100 g solution)			Core material (g/100 g wall material)	Surface tannin (g/100 g)	Total tannin recovery (g/100 g)	Encapsulation efficiency (g/100 g)

	GA	MD	NS				
MG-TE_25_	30	45	-	25	8.43^d^	70.7^ab^	64.8^a^
MG-TE_30_	28	42	-	30	10.7^d^	66.4^ab^	60.5^ab^
MG-TE_35_	26	39	-	35	11.2^d^	63.2^b^	56.1^b^
S-TE_15_	-	-	85	15	35.0^c^	75.1^a^	48.8^c^
S-TE_20_	-	-	80	20	53.4^b^	70.9^ab^	33.4^d^
S-TE_25_	-	-	75	25	63.9^a^	75.6^a^	27.7^d^
S-TE_30_	-	-	70	30	58.8^ab^	75.8^a^	31.1^d^
SEM	-	-	-	-	4.21	1.30	2.67
p-value	-	-	-	-	<0.0001	0.03	<0.0001

GA, gum arabic; MD, maltodextrin; NS, native starch; SEM, standard error of mean.

1) MG-TE, maltodextrin+gum arabic encapsulating acacia tannin extract (at 25%, 30%, 35% w/w); S-TE, native starch encapsulating acacia tannin extract (at 15%, 20%, 25%, 30% w/w).

a–d Means with different superscripts across a column are significantly different (p<0.05); treatments are expressed as mean and values are calculated from a minimum of four repeat batches.

**Table 2:** *In vitro* release kinetic parameters in acetate buffer media (pH, 5.6), of acacia tannin extract encapsulated with maltodextrin/gum-arabic and native starch (n = 3)

Items	Zero order, Q vs *t*	First order, ln (Q_0_-Q) vs *t*	Higuchi, Q vs $\sqrt{t}$	R^2^		

				Zero order	First order	Higuchi
ATE	Y = 2.861x+47.9	Y = −0.106x+1.78	Y = 27.1x+17.9	0.439	0.874	0.836
MG-TE	Y = 3.073x+39.4	Y = −0.090x+1.87	Y = 26.9x+10.5	0.543	0.952	0.924
S-TE	Y = 3.090x+37.8	Y = −0.080x+1.85	Y = 27.8x+7.6	0.491	0.819	0.865

Q_0_, Tannin to be released at zero time (mg); Q, amount of drug released at time t; t, time in hours; ATE, acacia tannin extract; MG-TE, acacia tannin extract encapsulated with maltodextrin+gum arabic; S-TE, acacia tannin extract encapsulated with native starch.

**Table 3:** *In vitro* gas production and fermentation parameters due to the addition of acacia tannin extract encapsulated with native starch or maltodextrin/gum arabic on *Eragrostis curvula* hay and total mixed ration feeds

Treatment1)	Gas production (mL/g DM)				Methane (mL/g DM)				24 h methane (%)	Gas production kinetics2)		pH	NH_3_-N (mM)
		
	2 h	4 h	12 h	24 h	2 h	4 h	12 h	24 h		b	c		
Eragrostis hay substrate													
Control (C)	12.0^bc^	19.0^cd^	27.5^c^	50.2^c^	1.06^bc^	1.81^cd^	2.77^b^	5.4^c^	10.6	125.8^c^	0.02^b^	6.93	11.5^a^
C+starch	17.0^b^	27.5^bc^	75.4^a^	167.7^a^	1.76^b^	3.04^bc^	7.90^a^	19.6^a^	11.5	184.2^b^	0.13^a^	6.84	10.5^ab^
C+S-TE	14.8^bc^	21.9^c^	66.5^ab^	162.5^a^	1.49^bc^	2.32^fg^	7.26^a^	18.8^a^	11.3	191.5^b^	0.13^a^	6.85	9.6^b^
C+maltodextrin-gum arabic	25.1^a^	38.6^a^	62.5^ab^	99.6^b^	2.85^a^	4.09^a^	6.51^a^	10.7^b^	10.5	225.5^a^	0.02^b^	6.72	10.5^ab^
C+MG-TE	23.6^a^	35.7^ab^	54.8^b^	87.1^b^	2.64^a^	3.95^a^	5.97^a^	9.5^b^	10.4	207.7^ab^	0.02^b^	6.85	10.0^ab^
C+ATE	9.9^c^	14.1d	18.8^c^	36.8^c^	0.90^c^	1.32^d^	1.86^b^	3.5^c^	10.7	100.7^d^	0.01^b^	6.91	10.1^ab^
SEM	1.75	2.65	6.21	14.91	0.23	0.31	0.69	1.81	0.18	9.64	0.02	0.07	0.7
p-value	0.005	0.002	0.001	<0.0001	0.003	0.001	0.002	<0.0001	0.38	0.0002	<0.0001	0.15	0.04
Total mixed ration substrate													
Control (C)	24.1^c^	42.1^b^	89.0^b^	154.2^d^	2.82^b^	4.30^b^	9.41^c^	17.0^d^	11.2	341.2	0.06	6.60	20.0
C+starch	25.5^c^	45.9^b^	129.4^a^	256.1^a^	2.88^b^	4.85^b^	14.3^a^	31.0^a^	12.1	336.5	0.06	6.34	19.8
C+S-TE	22.6^c^	39.5^b^	115.7^a^	236.7^b^	2.1^b^	3.97^b^	12.7^ab^	28.7^b^	12.0	288.3	0.05	6.68	18.4
C+maltodextrin-gum arabic	33.5^ab^	59.2^a^	124.2^a^	193.6^c^	3.47^ab^	5.82^ab^	13.0^ab^	22.3^c^	11.4	298.22	0.04	6.78	19.4
C+MG-TE	38.6^a^	63.2^a^	123.4^a^	184.4^c^	4.66^a^	7.12^a^	135^a^	21.4^c^	11.4	250.3	0.05	6.72	18.6
C+ATE	28.3^bc^	45.8^b^	90.1^b^	146.7^e^	3.13^ab^	5.34^ab^	10.7^bc^	18.0^d^	11.9	199.5	0.12	6.85	18.8
SEM	1.92	2.96	5.49	12.1	0.28	0.37	0.61	1.59	0.12	26.8	0.01	0.08	0.8
p-value	0.007	0.004	0.001	<0.0001	0.07	0.03	0.007	<0.0001	0.13	0.53	0.68	0.11	0.11

DM, dry matter; SEM, standard error of mean.

1) S-TE, acacia tannin extract encapsulated with native starch; MG-TE, acacia tannin extract encapsulated with maltodextrin+gum arabic; ATE, acacia tannin extract.

2) b, gas production (GP) from the insoluble but slowly fermentable fraction of substrate (mL); c, the rate of gp from insoluble fraction per hour.

a–e Means with different superscripts across same column are significantly different (p<0.05).
